# Findings from healthcare-associated infections data validation attestation in California general acute-care hospitals

**DOI:** 10.1017/ash.2022.183

**Published:** 2022-05-16

**Authors:** Nadia Barahmani, Andrea Parriott, Erin Epson, Genie Tang, N. Neely Kazerouni

## Abstract

**Background:** Accurate and complete hospital healthcare-associated infection (HAI) data are essential to inform facility-level HAI prevention efforts and to ensure the validity and reliability of annual public reports. We implemented a validation attestation survey to assess and improve the HAI data reported by California hospitals via NHSN. **Methods:** The California Department of Public Health (CDPH) HAI Program invited all 401 general acute-care hospitals in California to participate in an annual HAI validation attestation survey in 2021. The survey was designed to be completed by the person with primary responsibility for HAI surveillance and reporting consistent with NHSN protocols and California laws. Survey questions addressed HAI reporting knowledge and practices and surgical procedures performed, and they included 3 hypothetical scenarios evaluating hospital application of HAI surveillance, decision making, and reporting methods. **Results:** We received responses from 345 hospitals (86%). For the 3 hypothetical scenarios, 171 hospitals (49.6%) correctly answered all 3 questions, 110 hospitals (31.9%) answered 2 questions correctly, 52 (15.1%) hospitals answered 1 question correctly, and 12 hospitals (3.5%) answered zero questions correctly. We did not detect a statistically significant association between facility type (ie, acute-care hospital, critical access hospital, long-term acute-care hospital, or rehabilitation hospital or unit) and the probability of getting all questions correct (Fisher exact *P* = .42). Of the 303 hospitals (88.0%) that perform at least 1 of the 28 surgical procedures reportable in California, 269 (88.8%) apply CDPH-recommended postoperative ICD-10 diagnosis flag codes to identify records that might indicate a possible surgical site infection (SSI). Moreover, ~289 (84.0%) hospitals confirmed that someone at their facility reviews CDPH quality assurance–quality control reports to verify the accuracy and completeness of their hospital’s reported HAI data. In 321 hospitals (93.0%) decisions about which infections are reported to NHSN are made solely by the infection preventionists or hospital epidemiologists, who are thoroughly familiar and follow NHSN protocol, definitions, and criteria. **Conclusions:** Most hospitals reported following best practices for evaluating records for SSIs; however, only half responded correctly to all 3 hypothetical scenarios. Our results highlight the need for ongoing education on HAI surveillance, decision making and reporting methods, and external HAI data validation in hospitals. This survey could serve as a model for other states that work with hospitals to improve HAI surveillance data and to ensure the integrity of public reports. Future research will link the results of this survey to NHSN validation audits.

**Funding:** None

**Disclosures:** None

